# Supplementary material for: The folded X-pattern is not necessarily a statistical signature of decision confidence
Source: PLoS Comput Biol. 2019 Oct 21;15(10):e1007456. doi: 10.1371/journal.pcbi.1007456 (PMC6822779; doi:10.1371/journal.pcbi.1007456)
Supplement: S1 Appendix — (DOCX) [file pcbi.1007456.s001.docx]

**S1 Appendix. Derivation of the formula of objective confidence according to the standard model**

According to the standard model, it is assumed that an observer selects a choice $\vartheta\in\left\{ -1,1 \right\}$ about the identity I $\in\left\{ -1,1 \right\}\mathrm{of}$stimulus $d$. The identity equals the sign of d, which is sampled in each trial either from a discrete set or from a continuous distribution. The accuracy $A\in\left\{ 0,1 \right\}$ of the choice is defined to be 1 if $\vartheta=I$ and 0 otherwise. Observers cannot perceive $d$directly; instead, observers make their choices based on the sensory evidence e_I_, a noisy estimate of $d.$

Given the model specification, the posterior probability of being correct given the sensory evidence $p(A=1|e_{I})$ can be calculated as the posterior probability that identity is the same as the selected choice option, given the sensory evidence. In the following, we consider the case that the observer decides that the identity is 1; formulae for the decision that the identity is -1 can be derived just in the same way.

According to Bayes’ rule, $p(I=1|e_{I})$ can be calculated as:

| $\overset{\mathrm{posterior}}{\overbrace{p(I=1\vert e_{I})}}=\frac{\overset{\mathrm{prior}}{\overbrace{p(I=1)}} \times\overset{\mathrm{likelihood}}{\overbrace{p(e_{I}\vert I=1)}}}{\underset{normalisation constant}{\underbrace{p(e_{I})}}}$ | (3) |
| --- | --- |

Based on the law of total probability, the normalization constant $p(e_{I})$ can be expressed as:

| $p\left( e_{I} \right)=\sum_{j} p\left( I=j \right)\times p\left( e_{I} \vert I=j \right)$ | (4) |
| --- | --- |

For the purpose of the present analysis, we assumed that the two choice options are equally likely, i.e. the prior probabilities $p\left( I=-1 \right)$ and $p\left( I=1 \right)$ are both 0.5. Therefore, (3) and (4) can be combined and simplified to:

| $p\left( I=1\vert e_{I} \right)= \frac{p\left( e_{I} \vert I=1 \right)}{\sum_{j} p\left( e_{I} \vert I=j \right)}$ | (5) |
| --- | --- |

If $d$ is sampled from a finite set of n elements, the denominator of the fraction in (5) can be expressed as a sum of the likelihood of the sensory evidence conditioned on d over all possible values of d weighed by the probability of the specific $d$. For the numerator, the sum takes into account only positive values of d:

| $p\left( I=1\vert e_{I} \right)= \frac{\sum_{k, d_{k} > 0} p\left( d_{k} \right) p\left( e_{I} \vert d_{k} \right)}{\sum_{k} p\left( d_{k} \right) p\left( e_{I} \vert d_{k} \right)}$ | (6) |
| --- | --- |

If $d$ is sampled from a continuous distribution instead, numerator and denominator of the fraction in (4) can be expressed as integrals over d:

| $p\left( I=1\vert e_{I} \right)= \frac{\int_{0}^{\infty} p\left( d \right)p(e_{I}\vert d)dd}{\int_{-\infty}^{\infty} p\left( d \right)p(e_{I}\vert d)dd}$ | (7) |
| --- | --- |

In (7), *d* denotes the differential, while d denotes the stimulus.
