# Supplementary material for: The folded X-pattern is not necessarily a statistical signature of decision confidence
Source: PLoS Comput Biol. 2019 Oct 21;15(10):e1007456. doi: 10.1371/journal.pcbi.1007456 (PMC6822779; doi:10.1371/journal.pcbi.1007456)
Supplement: S2 Appendix — (DOCX) [file pcbi.1007456.s002.docx]

# S2 Appendix. Derivation of the formula of objective confidence according to the general model.

According to the general model, the observer is presented with a series of stimuli characterised by two features, the identity $I\in\left\{ -1,1 \right\}$ and discriminability d. It is assumed that observers in each trial make a choice $\vartheta\in\left\{ -1,1 \right\}$ about the identity $I\in\left\{ -1,1 \right\}$ of the stimulus. The accuracy $A\in\left\{ 0,1 \right\}$ of the choice is 1 if $\vartheta$ = $I$ and 0 otherwise. The decision is based on sensory evidence, which involves evidence about the stimulus strength $e_{d}$, which depends only on $d$, and evidence about the identity $e_{I}$, which depends on d and on I.

Given the model specification, the posterior probability of being correct given the sensory evidence $p(A=1|{(e}_{d},e_{I}))$ can be calculated as the posterior probability that the identity I of the stimulus is the same as the selected choice option, given the sensory evidence. In the following, we consider the case that the observer decides that I is 1; the formula for the choice that I is -1 can be derived analogously.

According to Bayes’ rule, $p(I=1|(e_{D},e_{I}))$ can be calculated as:

| $\overset{\mathrm{posterior}}{\overbrace{p(I=1\vert{(e}_{I},e_{d}))}}=\frac{\overset{\mathrm{prior}}{\overbrace{p(I=1)}} \times\overset{\mathrm{likelihood}}{\overbrace{p({(e}_{I},e_{d})\vert I=1)}}}{\underset{normalisation constant}{\underbrace{p(e_{I},e_{d})}}}$ | (8) |
| --- | --- |

As we assumed again that the two choice options are equally likely and thus the prior probabilities of both identities are the same, formulae (8) can be simplified analogously to formula (5):

| $p\left( I=1\vert(e_{I},e_{d}) \right)= \frac{p\left( {(e}_{I},e_{d})\vert I=1 \right)}{\sum_{j} {p((e}_{I},e_{d})\vert I=j)}$ | (9) |
| --- | --- |

If $d$ is sampled from a discrete set of elements, numerator and denominator in (8) can be expressed as a sum of likelihoods of the sensory evidence conditioned on $d$ over the different values of d. The sum is weighed by the probability of each specific $d$:

| $p\left( I=1\vert(e_{I},e_{d}) \right)= \frac{\sum_{k} p\left( d_{k} \right)\times p((e_{I},e_{d})\vert(I=1, d_{k}))}{\sum_{j,k} p\left( d_{k} \right) \times p((e_{I},e_{d})\vert(I=j,d_{k}))}$ | (10) |
| --- | --- |

Given the case that d is sampled from a continuous distribution, $p\left( I=1|{(e}_{I},e_{d}) \right)$ can be obtained by integration:

| $p\left( I=1\vert e_{I},e_{d} \right)= \frac{\int_{0}^{\infty} p\left( d \right)\times p({(e}_{I},e_{d})\vert(I=1, d))dd}{\int_{0}^{\infty} \sum_{j} p\left( d \right)\times p((e_{I},e_{d})\vert(I=j,d))dd}$ | (11) |
| --- | --- |

Again, d denotes the differential, while d denotes the discriminability of the stimulus. As we assume that $e_{I}$ and $e_{d}$ are stochastically independent when the stimulus strength d is controlled, the likelihood $p({(e}_{I},e_{d})|(I,d))$ can be calculated as:

| $p\left( {(e}_{I},e_{d}) \vert(I, d) \right)= p\left( e_{d}\vert d \right)\times p(e_{I}\vert(d,I))$ | (12) |
| --- | --- |

We insert formula (12) into (10) to calculate $p\left( I=1 | e_{i},e_{D} \right)$ in the discrete case.

| $p\left( I=1\vert(e_{I},e_{d}) \right)= \frac{\sum_{k} p\left( d_{k} \right) \times p\left( e_{d}\vert d_{k} \right)\times p(e_{I}\vert(d_{k},I=1))}{\sum_{j,k} p\left( d_{k} \right) \times p\left( e_{d}\vert d_{k} \right)\times p(e_{I}\vert{(d}_{k},I=j))}$ | (13) |
| --- | --- |

Finally, we insert formula (12) into (11) to calculate $p\left( I=1 | {(e}_{I},e_{d}) \right)$ in the continuous case.

| $p\left( I=1 \vert e_{I},e_{d} \right)=\frac{\int_{0}^{\infty} p\left( d \right)\times p\left( e_{d}\vert d \right)\times p(e_{I}\vert(d,I=1))dd}{\int_{0}^{\infty} \sum_{j} p\left( d \right)\times p\left( e_{d}\vert d \right)\times p(e_{I}\vert(d,I=j))dd}$ | (14) |
| --- | --- |
